# Leading Learning Health Systems in the Asia Pacific: A Mixed‐Method Evaluation of Health Professionals' Experiences in a Longitudinal Leadership Program

**DOI:** 10.1002/lrh2.70109

**Published:** 2026-08-07

**Authors:** Lichin Lim, Carolyn Van Heerden, Amy Gray

**Affiliations:** ^1^ Department of Paediatrics University of Melbourne Parkville Victoria Australia; ^2^ Royal Children's Hospital Melbourne Parkville Victoria Australia

**Keywords:** capacity building, continuous learning culture, leadership, learning health system

## Abstract

**Introduction:**

The Learning Health System (LHS) concept is gaining traction in high‐income countries, yet its implementation in low‐ and middle‐income countries (LMICs) remains limited with few initiatives offering practical strategies tailored to these contexts. The Leading a Learning Health System (LLHS) Program, grounded in LHS principles, aimed to build leadership capacities among health professionals and leaders from five Asia‐Pacific LMICs. This study explored participants' learning experiences and applications in their context.

**Methods:**

A mixed‐method longitudinal study was conducted (13‐months), informed by social constructivism and Kirkpatrick's evaluation framework. Surveys and semi‐structured interviews were conducted after the intensive course (Survey A and Interview A) and 1 year later (Survey B and Interview B). Quantitative data were analyzed descriptively, and qualitative data were analyzed through inductive content analysis.

**Results:**

Thirteen participants (13/20, 65%) completed Survey A. All (100%) rated the program and cross‐country network as “high value.” Eleven participants (11/20, 55%) completed Survey B. All participants reported applying program learnings regularly on most days (6/11, 55%) or most weeks (5/11, 45%). Nine participants (82%) reported influencing team culture and five (45%) reported influencing organizational change. Qualitative analysis of 20 Interview A and 10 Interview B identified four themes: (1) developing a systems leadership identity, (2) translating leadership intent into practice, (3) contextual challenges to applying learnings, (4) resilience via adaptive leadership capacity and programmatic support.

**Conclusion:**

This study demonstrates how a longitudinal leadership program based on the LHS framework supported LMIC health professionals to develop systems leadership identity, foster learning culture, and cultivate adaptive, distributed leadership to build resilience in resource‐limited settings. The variability of health information systems in LMICs should not be seen as a barrier to implementing LHS, as improvement can begin with the effective use of locally available data. These findings can inform future capacity‐building initiatives to advance LHSs in LMICs.

## 1 Introduction

Health systems are complex, adaptive social systems that respond to evolving health, social, and political contexts [1, 2]. Managing these complex and evolving health needs poses challenges for all health systems [1]. These are particularly evident in low‐ and middle‐income countries (LMICs) due to high disease burdens, rapid population growth, and limited resources [3, 4, 5].

The Learning Health System (LHS) concept offers a framework for continuous improvement of health systems through iterative learning cycles [6], as illustrated by the Friedman model where practice generates data, data generates knowledge, and knowledge is applied back into practice [7]. While digitalisation of health systems initially drove the LHS concept by enabling the sharing of large datasets for data‐driven innovation, social and cultural dimensions are now recognized as equally critical [8]. A recent review of various LHS frameworks suggests broad consensus around Friedman's cycle as the core of the LHS, supported by six interdependent domains adapted from the Institute of Medicine: science and informatics, continuous learning culture, incentives, patient–clinician partnerships, structures and governance, and equity and ethics [6, 9, 10].

While the LHS framework has gained traction in high‐income countries, its application in LMICs remains limited [3, 5]. A systematic review of 219 LHS articles found that only 3% were from middle‐income countries and none from low‐income countries [9]. Several factors contribute to this gap. The global LHS discourse has been shaped predominantly by high‐income countries, overlooking the specific needs of LMICs [3, 5]. Rigid bureaucracies, short‐term funding, fragmented data practices, or a lack of mature health information systems, operational pressures, and a lack of institutional structures for iterative learning limit the implementation of LHSs in LMIC [3, 5, 11]. There is also limited shared understanding of what constitutes an LHS or how it should function in LMIC, often relating to a disconnect between policies and realities [12]. When health systems fail to engage in systematic learning from past initiatives, they risk repeating mistakes, leading to the failure of policies and programs [4]. Therefore, embedding learning to strengthen health systems is especially critical in LMIC contexts.

Institutionalizing learning at all levels of the health system—individual, team, organizational, and cross‐organizational—is required for an effective LHS [1]. Yet, many LMIC learning activities are undervalued and underfunded as health systems lack the capacity to support such learning [1, 3]. Studies from LMICs highlight the importance of leadership in building relationships and collaboration. They also advocate for distributed leadership models and participatory approaches [12, 13, 14, 15, 16, 17]. While there are calls for long‐term investments in leadership development and LHS culture [13, 15], few initiatives offer practical strategies to build this capacity [5, 18].

We developed and implemented a year‐long Leading a Learning Health System (LLHS) program based on the LHS framework to build LHS‐informed leadership capacities among healthcare leaders from LMICs.

This study aimed to explore the experiences of health professionals from five Asia‐Pacific LMICs who participated in the LLHS Program, in terms of their own learning and development, and the application of learnings in their context.

## 2 Methods

### 2.1 Program Development and Delivery

The LLHS program was developed by an academic Australian research group working in the Asia Pacific to test the hypothesis that introducing the LHS concept with emphasis on leadership, learning methods and data use could support new ways of work and responding to health challenges for health professionals in the region—both independently and in collaboration together. Program development was informed by established LHS frameworks, particularly the WHO Alliance for Health Policy and Systems report which describes the levels of learning (individual, teams, organization), with data as a critical component alongside enabling factors such as leadership [1]. The study team had significant prior experience in leadership development, clinical education and designing capacity‐building programs in the Asia Pacific.

The LLHS program commenced with a two‐week, in‐person intensive course in Australia, removing participants from the distractions of daily work. Table 1 outlines the curriculum which integrates three interrelated components: “Leading” to build leadership capability, “Learning” at individual and team levels, and “Helping system learn” focusing on the use of data for continuous improvement.

**TABLE 1:** Overview of the LLHS curriculum.

Component	Purpose	Key content areas	References
Leading	Build leadership capability to support system change	Effective leadership practice, emotional intelligence, team functioning, conflict management	[19, 20, 21, 22]
Learning	Foster learning at individual and team levels	Psychological safety, feedback, coaching, debriefing and reflective practice	[23, 24, 25, 26]
Helping system learn	Build capability in using data to support continuous improvement	Identifying problems in health systems, recognizing different sources of data, using data to inform iterative improvement cycles	[8, 27, 28]

By the end of the 2‐week intensive, participants developed individual or group action plans for self‐selected improvement initiatives to implement in their local settings. Ongoing support was provided through a virtual learning community that was maintained via a messaging group and three online forums over the year. The community provided a space for peer learning to sustain engagement.

On return to their home countries, participants received continued support from the program faculty, including in‐country visits to co‐deliver LLHS courses with participants' local networks.

### 2.2 Participant Recruitment

Participants were purposefully selected for the LLHS Program through nomination by their employing organizations, which had established partnerships with the Australian team. All 20 participants were invited to take part in this study. There were no exclusion criteria.

### 2.3 Study Design and Data Collection

This study adopted a social constructivism lens, which recognizes that knowledge is co‐constructed through social interaction [29]. The research explores participants' experiences of engaging with the LLHS Program, acknowledging that these experiences are influenced by their roles, peer relationships, and diverse health system environments. This worldview places participants at the center of the meaning‐making experience and as active interpreters of their learning journeys [30].

This study applied a parallel mixed‐method longitudinal design over 13 months (November 2023 to December 2024), guided by Kirkpatrick's evaluation framework [31, 32]. Quantitative and qualitative data were collected at the same timepoints, with qualitative findings providing depth and triangulation to enhance credibility of results [32]. This approach allowed exploration of how participants' responses evolved over time and whether learning was sustained [33]. Table 2 outlines the study design and data collection across the two timepoints, including evaluation focus and methods.

**TABLE 2:** Overview of study design and data collection.

Timing	Evaluation focus with Kirkpatrick levels	Data collection via survey^a^	Data collection via interview^a^
*Timepoint 1* Immediately post‐intensive course (November 2023)	Perceived value of the program (Level 1 Reaction) Key learning and intended changes to practice (Level 2 Learning)	Survey A Likert scale Open‐ended questions	Interview A (in‐person, semi‐structured) conducted by two researchers (LL and JH)
*Timepoint 2* At 12‐month follow‐up (November–December 2024)	Application of learning, confidence, influencing factors (Level 3 Behavior) Perceived organizational impact (Level 4 Results)	Survey B Likert scale Multiple choice questions Open‐ended questions	Interview B (online, semi‐structured) conducted by one researcher (LL) through video call

^a^ Survey and interview questions are outlined in the Appendix A.

All survey data was collected and managed using data capture tools (REDCap hosted by The University of Melbourne) with survey completion implying consent. Verbal consent was obtained at the start of each interview, which was audio‐recorded, transcribed using transcription software (Descript), then manually checked and anonymized by a researcher (LL).

### 2.4 Data Analysis

Quantitative data from each timepoint were analyzed using descriptive statistics, including numbers and percentages. No inferential statistics were used, as the surveys contained different questions and were intended to capture perceptions at two distinct timepoints.

Qualitative data from open‐ended survey responses and semi‐structured interviews were analyzed using inductive content analysis [34], with a combination of consensus and split coding. Initially, three researchers (LL, AG, and CvH) individually coded the same two transcripts from Interview A. The research team then met to discuss, compare, and refine codes and created a preliminary codebook. Two researchers (LL and CvH) subsequently coded another five transcripts from Interview A. While refining codes, the research team recognized the potential insights to be gained from examining the trajectory of responses between the first and second interview sets. The two researchers subsequently coded two transcripts from Interview B independently. The research team then met again to compare interpretations, discuss any new codes that had emerged, or modify existing codes. Following this, a single researcher (LL) independently coded the remaining transcripts from both interview sets. Periodic discussions were held to ensure continued alignment with coding and consistency in interpretation. Themes and subthemes were developed iteratively.

This study was approved by The Royal Children's Hospital Melbourne Human Research Ethics Committee (HREC/109282/RCHM‐2024).

## 3 Results

A total of 20 participants were invited to participate in both surveys and interviews. Table 3 shows the number and demographics of participants who contributed to each data set.

**TABLE 3:** Participants' demographics, characteristics and study participation.

Participants	*n* (%)	
Total	20 (100)	
Country of origin		
– Laos	– 4 (20)	
– Indonesia	– 7 (35)	
– Vietnam	– 3 (15)	
– Fiji	– 4 (20)	
– Vanuatu	– 2 (10)	
Sex		
– Female	– 16 (80)	
– Male	– 4 (20)	
Clinical profession		
– Medical	– 17 (85)	
– Nursing	– 2 (10)	
– Allied health	– 1 (5)	
Seniority		
– Self‐perceived as senior leader	– 8 (40)	
– Self‐perceived as mid‐level leader	– 3 (15)	
– Self‐perceived as junior leader	– 9 (45)	
Study participation		
– Surveys	Survey A – 13 (65)	Survey B – 11 (55)
– Interviews	Interview A – 20 (100)	Interview B – 10 (50)
Country of origin		
⚬ Laos	⚬ 4 (20)	⚬ 1 (5)
⚬ Indonesia	⚬ 7 (35)	⚬ 3 (15)
⚬ Vietnam	⚬ 3 (15)	⚬ 3 (15)
⚬ Fiji	⚬ 4 (20)	⚬ 2 (10)
⚬ Vanuatu	⚬ 2 (10)	⚬ 1 (5)

Thirteen participants (13/20, 65%) responded to Survey A (Table 4). All (100%) regarded the 2‐week intensive course as “high value” and stated they would recommend the program to other colleagues in their countries. Similarly, all participants (100%) rated interaction with colleagues from other countries as “high value.”

**TABLE 4:** Survey A participant ratings of LLHS intensive course (*n* = 13).

Survey item	High value, *n* (%)
Overall value of the LLHS course	13 (100)
Would recommend the course to colleagues	13 (100)
Value of interaction with international peers	13 (100)
Value of the “Leading” component	12 (92)
Value of the “Learning” component	13 (100)
Value of the “Helping systems learn” component	13 (100)

One year later, 11 participants (11/20, 55%) completed Survey B (Table 5). Six participants (6/11, 55%) reported applying learnings from the LLHS Program in their work on most days, and 5 participants (5/11, 45%) reported applying learnings on most weeks. All participants felt very confident (3/11, 27%) or confident (8/11, 73%) in applying the knowledge and skills gained through the program. In terms of perceived influence on practice, most participants (9/11, 82%) reported being able to influence their team's culture towards continuous learning and improvement. Fewer participants (5/11, 45%) indicated they had been able to influence change at the organizational level.

**TABLE 5:** Survey B findings at 12‐month follow‐up (*n* = 11).

Survey item	Response	*n* (%)
Frequency of applying LLHS learnings in practice	Most days	6 (55)
	Most weeks	5 (45)
Confidence applying knowledge and skills	Very confident	3 (27)
	Confident	8 (73)
Influence on team culture	Yes	9 (82)
	Not sure	2 (18)
Influence on organization	Yes	5 (45)
	No	3 (27)
	Not sure	3 (27)
Barriers to implementation^a^	Lack of time	8 (73)
	Insufficient resources	5 (45)
	Organizational constraints	3 (27)
	Resistance from colleagues	2 (18)

^a^ Multiple responses permitted for Barriers to implementation.

**FIGURE 1:**
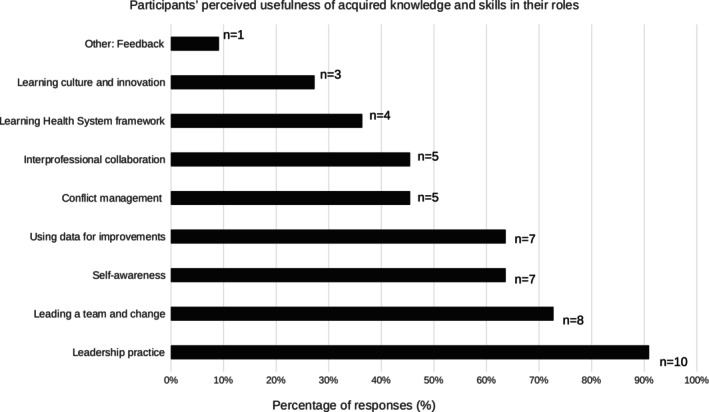
Survey B: Proportion of responses from participants indicating which specific knowledge or skills from the program were most useful in their roles.

The most cited knowledge and skills from the program that participants found useful were leadership practices, leading a team and change, self‐awareness and using data for improvement (Figure 1). When asked to identify which barriers they encountered when trying to implement change, lack of time was cited more frequently (8/11, 73%), followed by insufficient resources (5/11, 45%) and organizational constraints (3/11, 27%).

**FIGURE 2:**
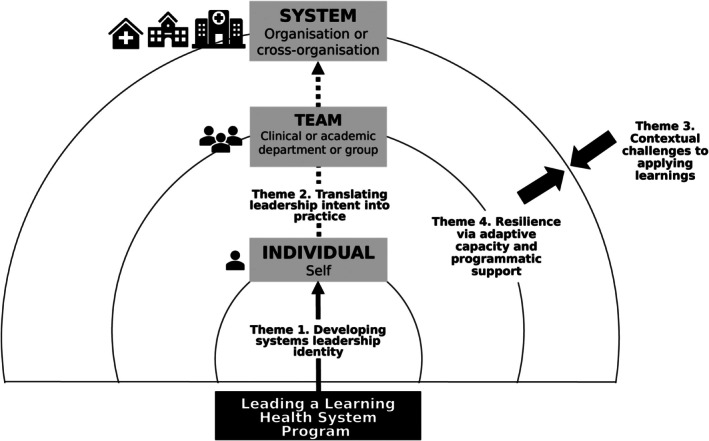
Four themes derived from qualitative data.

These quantitative findings were reinforced and elaborated by qualitative interview data at both time points, with four themes identified (Figure 2) and discussed below with illustrative quotes in Table 6.

**TABLE 6:** Illustrative quotes for the four themes found in the qualitative data.

Theme/subtheme	Examples quotes
*Theme 1. Developing a systems leadership identity*	
*Reframing leadership identity*	I am not only a clinician and a lecturer… but also have the responsibility to our community. Not only community in pediatrician or young doctor, but also our stakeholders in the district or provincial health office, hospital, private health centers. P07, Indonesia, Interview A
	Leadership and learning health system… these two are very close to each other. If leadership is good or correct, then the health system is learning. If the leadership is not open to leading change, then you cannot make the health system become learning health system. The health system maybe stable, but not improving with time. P06, Vietnam, Interview A
	I'm basically the clinician… Before I want to see the clinic, but after I attended the workshop, I feel I should not go to the clinic, I should focus on leadership learning. We (senior clinicians) don't need the clinical training, but we need the system training… to learn the system. P09, Indonesia, Interview B
	I have to implement Learning Health System in our department, but it can bigger. I joined in the Indonesian Pediatric Society, I joined the local district health office. I can broaden my influence of learning and leadership in those areas also. P01, Indonesia Interview B
	Insight to be a leader instead of manager; not just focus on what is the target but also the process to achieve, with warmth but systematic. Survey B
	The LLHS program is very different from other leadership programs. It gives us time to reflect on ourselves and us in leadership roles. It gives us reasons and ways/ideas to change ourselves to have a better team in a learning health system. Survey B
*Equipping self with leadership tools*	It's so easy to implement what we learn here and to correlate with our work. In this course I got the depth… it happened very clearly, structured systematically and a different way to change people. P18, Indonesia, Interview A
	I will apply myself in conflict solutions. Before I try to avoid conflict. But we have learned from here… we have many different ways that we can apply in different situations of conflict. Very useful. P17, Laos, Interview A
	We can do a small study in our workplace. P13, Vanuatu, Interview A
	Do not ignore any problem. I will try to process a small data we have to make a study that can be used to improve the services. Survey A
	Listen and communicate to understand others and know that people have their own characteristics. So we should develop others' strengths to have a strong team. Survey A
	The program in Melbourne is very useful for us. The program integrated theory with practice, making theory understandable and easy to remember and to understand deeply, and then it can be used in reality, in practice. Knowledge was taught… with a very good method. After the program we have more skills to act on. The course contains a lot of good materials and can be used in our own context. P06, Vietnam, Interview B
*Theme 2. Translating leadership intent into practice*	
*Leading through trust, connection and recognition of strengths*	Connecting and then leading. Make them have trust in yourself and not to let them work alone…. We have to support them and sometimes, we have to do it together, as a team. P10, Laos, Interview B
	The young leaders (P03 and P08) know how to choose people. They know people, they know their staff and what they want. They know how to encourage people to work in a busy environment, to do many tasks at the same time. P06, Vietnam, Interview B
	The training has provided me with a better tool for assessment. Identifying team members' strengths and weaknesses and working with them, and the different personalities… it helped us to work together. It was much better teamwork than what we had with the previous team. P15, Fiji, Interview B
	The team dynamics, the dysfunctions of the team that we talked about… unless and until you are sensitized to those things, you will not be able to address it. So, a lot of things might be happening at your team level, but you will just not be aware because you have no knowledge. P12, Fiji, Interview B
	There are a huge of benefits LLHS program… First, it changed the way we communicate to each other. To build a strong team at work, we start with trust then lead. We created a safe and supportive environment at work. Second, we changed the way we give feedback to help others improve, not criticize others' mistakes. We have discussions to learn from each other. Survey B
*Fostering a learning culture*	Listening to people, encouraging people to talk, creating a safe environment for people to express all ideas on constructive feedback for the hospital unit. P06, Vietnam, Interview B
	I am able to always have a coaching attitude and be able to always take a step back. Have a debriefing session. These are small tools that I use, which really make the difference. P19, Vanuatu, Interview B
	I build the system that people should resolve their conflict, not run from the conflict, not fear of the conflict. Develop that culture to make our situation more open, fair. P09, Indonesia, Interview B
	I'm just so happy that they can openly disagree now. If they don't agree with things, they will tell us, and it's the only way of improving. P12, Fiji, Interview B
	Conducting reflections on every incident and complaint that occurs as a learning opportunity… not only improved our processes but also strengthened team communication and accountability. The program shifted my mindset from being a problem‐solver for my team to empowering them to take ownership of challenges and solutions… fostered a more engaged and proactive culture within the PICU unit. P02, Indonesia, Interview B
	90% of my team members are starting to become aware of the problems in the PICU. Previously the problem was known only to primary nurses and team leaders. My team seemed more responsive to patient problems and non‐patient problems, such as compliance in filling in patient data, recording the use of medical equipment. Survey B
*Theme 3. Contextual challenges to applying learnings*	
*Environmental factors*	Another challenge is time. Balancing day‐to‐day operations while trying to implement improvements can be difficult, as both require significant attention and effort… Sometimes, due to other commitments, the monitoring process becomes less optimal. P02, Indonesia, Interview B
	One thing that I didn't do, and I have been dragging this for too long, is to be able to do a clinical audit. I've got tools that I don't use. I'm telling myself I will make time, I will make time, but I don't have time. Like this month, I am the only one on call every day… That's our situation, you're on by yourself until God knows when the next help will come. P19, Vanuatu, Interview B
	In one big city, they think that their local government has already improved primary health care. They don't see the need to improve or collaborate with primary health care… probably they don't notice the data. P09, Indonesia, Interview B
	With every change, we see a new person (senior leader) coming up with their own vision. We develop a new strategic plan, new direction and new way of doing things, with very little time to implement. P12, Fiji, Interview B
	Even though it is not big data, it is only data from one unit or one department, we still can use it. P05, Indonesia, Interview B
	We want to gather then share the data to show provinces they still have child mortality. Then use the data to modify or personalize program for each province. After we have this baseline data capture, we can have more targeted intervention, probably more localized—smaller, but more intensive. P09, Indonesia, Interview B
*Social influences*	In Asian culture, we fear conflict. Mostly the conflict is not resolved. It's underground. P09, Indonesia, Interview B
	In our culture, if you are junior, you have to listen to the senior, even if your ideas are correct. For us in the healthcare system, a lot of the work is from the ministry and from the different levels of hierarchy, often you're not heard as much. P10, Laos, Interview B
	We have to follow the Office of Training and Education. We cannot change because it's already prepared or determined. P03, Vietnam, Interview B
	It's quite difficult to lead when you are a middle line manager, so you're trying to become a link between those people who are up there and those people who are down there working to get the job done. P12, Fiji, Interview B
*Theme 4. Resilience via adaptive leadership capacity and programmatic support*	
*Adaptive leadership capacity at different levels*	The program helps us, empowers us, especially to lead in a challenging environment like Vanuatu. We are very resource limited in all aspects, yet this program is able to teach us to look at ways we can learn from our situation and keep leading the people to achieve the vision and mission of the hospital. P19, Vanuatu, Interview B
	If you've connected well with your team and you've got a good team, you can always depend on them to get the job done. You can always effect change at your level and that's where we are finding stability right now when everything else is haywire. I think we've become resilient to those (senior) leadership changes, understanding that those things are outside of our control. I won't say that we have a lot of support from people higher in the hierarchy to affect those changes. But I don't think that really affects us much because we can lead by example in the small things that we are doing. P12, Fiji, Interview B
	So we don't leave anyone behind. We want everyone in our team. We have to learn from members of our team. We have to solve the program together. We try to work for our society, not for the individual person. P10, Laos, Interview B
	In my country, we have so many problems with the healthcare system. The most challenging they think is that we lack funding or facility. I want to switch the mindset to not focusing on the facility or tools, but focusing on the human resources, especially the thought and confidence in leadership, so that in whatever situation we still can improve our healthcare facility. P09, Indonesia, Interview B
	I (senior leader) have many roles and may not have time to teach my staff. It is useful to send them to a condensed course so that they have tools such as coaching, debriefing, building trust and connection when they need to lead the team. The program already helped younger leaders… change the environment in our unit, our hospital, our teaching and the way they approach work. P06, Vietnam, Interview B
*Sustaining momentum through programmatic support*	When we want to do something different… it's not easy, especially for junior staff like me to encourage the senior staff. I can discuss with P7 and P5. P7 is quite senior, we can discuss how to improve through a workshop at the department level. P01, Indonesia, Interview B
	It is good to have cross‐learning system in our region. While listening to other countries, we can learn to solve the problem by seeing how other people solved the problem first… If we only see the high‐income countries… it seems very hard to achieve the goal. But if we see people in our region who have already succeeded in making improvements, it will encourage us to do the same. In developing countries like my country, we have limited resources. Sometimes I give up after we try. If they (other LMIC) can do something and prove it, there is no choice but we can do this. It's like recharging energy “this might happen if we try.” P09, Indonesia, Interview B
	If you are alone, maybe you think that is a big problem… But if you have connection, you can share… everyone around the world can support you, can help each other. They (other participants) are facing problems, and I'm not alone in what I'm also facing in terms of changes and hierarchy… gave me a bit more confidence on how I carry out my work. P10, Laos, Interview B
	We were able to connect and understand the problems that we have are common, and so there's no need to be discouraged that it is only us facing these issues. P12, Fiji, Interview B
	I would like to see the LLHS program being integrated into the curriculum for young leadership in my country. Survey B

### 3.1 Theme 1: Developing a Systems Leadership Identity

#### 3.1.1 Reframing Leadership Identity

Immediately post‐intensive, participants reflected on developing self‐awareness to critically evaluate their leadership behaviors and past experiences. Participants indicated moving beyond viewing themselves solely as clinicians, academics, or managers to adopt a broader systems leadership identity. They described gaining awareness of their role within complex health systems and recognized that leadership is a critical enabler for health system improvement. They reframed leadership from a top‐down authority to one grounded in collaboration, shared accountability, and mutual respect, as one participant quoted “to become a leader who is respected, not feared.” (P02, Indonesia, Interview B). Participants reported shifting their priorities, for example prioritizing leadership and systems training over clinical training or engaging more actively with external stakeholders.

#### 3.1.2 Equipping Self With Leadership Tools

Participants described the tools acquired through the program as relevant, easy to implement, and adaptable to their local contexts. A key learning area was developing emotional intelligence and social awareness when working across diverse roles and hierarchies, which could support more adaptive responses and improve conflict resolution in the workplace. Tools to encourage learning, such as reflective practice, coaching, and feedback, were frequently cited. Several participants recognized the value of using available local data and Plan‐Do‐Study‐Act (PDSA) cycles to drive small‐scale change. Participants' reflections demonstrated a strong sense of motivation and readiness to enact change, expressing clear intentions to apply their learning in practice.

### 3.2 Theme 2: Translating Leadership Intent Into Practice

#### 3.2.1 Leading Through Trust, Connection and Recognition of Strengths

Participants recognized trust and human connection as foundations of effective leadership, shifting their mindset from focusing on resource limitations to enabling human resources. Participants expressed a desire to “focus on team members, not on barriers*”* (P09, Indonesia, Interview A) and to “*nurture people”* (P06, Vietnam, Interview A). To build trust, participants described enacting leadership practices such as role modeling, showing vulnerability, sharing a clear vision, supporting personal development, and creating a safe work environment. One participant observed that when the work environment improved, staff “go out of their way, they work extra hours to get all these things done” (P12, Fiji, Interview B).

Participants reported a deeper understanding of their team members, making deliberate efforts to observe team dynamics and align responsibilities with individual strengths to enhance teamwork. They also described increased sensitivity to emerging team issues they were previously unaware of. One participant reflected on using self and social awareness to better support the team when dysfunction emerged.

#### 3.2.2 Fostering a Learning Culture

Participants described fostering a learning culture and growth mindset within their teams, with psychological safety as a foundation. Tools such as feedback, coaching, and debriefing were commonly used to facilitate non‐judgemental learning. Several participants described creating a safe environment for open disagreement and constructive conflict, while some used data to prompt team reflection—for example, reviewing data from incidents or complaints as a learning opportunity rather than punitive responses. Participants recognized that leadership enables team collaboration and collective problem‐solving, leading to improvements in work processes.

Participants reported tangible outcomes (summarized in Table 7), including achieving individual and team awards, implementing improvement initiatives, initiating cross‐departmental and cross‐institutional collaboration, and in some cases, influencing policy changes.

**TABLE 7:** Examples of local initiatives led by participants, including recognition of individual and team achievements.

Country	Participant	Examples of implementation and achievement
Fiji	P12	Developed and implemented new promotion criteria recognizing all professions Successfully advocated for equitable professional development funding policy for all staff Received international accreditation for two undergraduate programs Received Distinguished College Teacher Award and Distinguished College Leadership Award Team received Outstanding Achievement in Learning and Teaching Award and Staff Wellbeing Award
	P15	Initiated the Pacific Child Health Research Reference Group in collaboration with P04's team, aiming for 2025 launch
Indonesia	P09	Established a national research group and career pathway Implemented case‐based discussion program for primary healthcare workers across provinces Delivered LHS‐informed leadership training to Indonesian Pediatric Society and Ministry of Health
	P02	Introduced evidence‐based practice changes in PICU (e.g., prone positioning for ventilated patients) Created internal learning system using Google Classroom with fortnightly content Engaged PICU nursing staff to support hospital‐wide patient feedback initiative
	P01	Improved equality in academic staff teaching time Delivered LHS‐informed leadership training to Faculty of Medicine, Public Health and Nursing across departments
Vietnam	P06	Led Ministry of Health and WHO‐supported initiative where 15 of 30 NICU doctors provided outreach training and support at provincial hospitals Delivered LHS‐informed leadership training to University Faculty of Medicine and Pharmacy and various hospital departments
	P08	Introduced changes to NICU policies and procedures to enhance staffing and continuity of care. Examples include: ⚬ Nurse and doctor pairing model ⚬ Cross‐ward collaboration between NICU and postnatal services ⚬ Streamlined leave/absence process
	P03	Introduced and integrated mini‐CEX into undergraduate medical student curriculum
Laos	P10	Introduced flipped classroom approach in undergraduate curriculum Appointed President, Lao Youth Union and Vice‐President, Lao Dental Association, attributed to collaborative and shared decision‐making leadership approach
Vanuatu	P19	Delivered LHS‐informed leadership training to hospital nursing and medical teams

### 3.3 Theme 3: Contextual Challenges to Applying Learnings

#### 3.3.1 Environmental Factors

Participants' ability to sustain application of learnings was influenced by a range of environmental factors, with time and resource limitations most common. Participants struggled to balance routine responsibilities with improvement initiatives, with workforce shortages limiting capacity to implement improvement initiatives such as clinical audits. System fragmentations were described as a barrier, preventing collaboration across departments and institutions, while frequent changes in organizational leadership disrupted continuity.

Challenges related to data were relatively absent in participants' narratives. Some recognized the potential of using data to motivate or enhance collaboration in resource constrained settings. One noted the practical limitation on manual data collection where person time was not available. Another noted in the absence of mature electronic data systems, they had readily available data sources. Many participants, however, described practical ways to use data to support their improvement initiative.

#### 3.3.2 Social Influences

Social and cultural dynamics were perceived to influence participants' ability to apply their learnings. Many described ingrained norms favoring conflict avoidance, preventing team members from speaking up. In hierarchical contexts, participants indicated that authority and seniority often determined whose voices were heard, regardless of the value of contributions. Participants in more junior roles reported lacking role authority to enact change and, in some cases, a lack of psychological safety. It was stated that expressing divergent views could negatively impact one's reputation or lead one to “be victimized” (P12, Fiji, Interview B). Those in mid‐level managerial roles described feeling caught between the expectations of senior management and team members.

### 3.4 Theme 4: Resilience via Adaptive Leadership Capacity and Programmatic Support

#### 3.4.1 Adaptive Leadership Capacity at Different Levels

Participants described strategies to sustain leadership practices despite challenges. At an individual level, participants reported building resilience and adaptive capacity, drawing on program insights to remain motivated despite limiting conditions. Several highlighted the importance of having patience, recognizing not all problems can be solved and the need to focus on *“own setting”* (P12, Fiji, Interview B) and “learn from our situation” (P19, Vanuatu, Interview B) to make incremental improvements.

At the team level, participants described building team identity and resilience by forming value‐driven teams that could withstand external pressures. Collaborative problem‐solving was a recurring theme to foster shared ownership of challenges. At the organizational level, participants highlighted building local leadership capacity by empowering junior staff and supporting distributed leadership. Participants also described disseminating program learnings through informal discussions, structured training, and engagement with external stakeholders, including ministries of health and provincial hospitals.

#### 3.4.2 Sustaining Momentum Through Programmatic Support

Ongoing programmatic support was valued as a key source of motivation. Participants who attended with colleagues from the same organization or country found local peer networks reinforced learning and supported their implementation efforts.

Connections with international peers were also highly valued. Participants noted a strong sense of shared purpose and mutual understanding due to similar health system structures and challenges. Cross‐system learning was perceived to enable participants to “discuss, compare and contrast between our practices and systems” (P15, Fiji, Interview A). Hearing successful examples from similarly resource‐limited settings was particularly motivating, reinforcing participants' belief that change was possible in their own context.

Participants described that the program's virtual platforms of group messaging and online forums provided a safe space for shared problem‐solving and peer support. Some sought advice directly from peers. Many described the learning community as reducing isolation and increasing their confidence in navigating local challenges. One participant noted that having the program's faculty co‐deliver local training was helpful, as local leaders were more receptive to external facilitators.

## 4 Discussion

This study explores the experiences of health professionals from five Asia‐Pacific LMICs who participated in a longitudinal LHS leadership program. The program was highly valued and supported evolution leadership identify, application of learnings, and examples of local change over 1 year. Participants described a shift towards collaborative leadership with efforts to build local leadership capacity across levels. The program's cross‐country learning community provided social and technical support to sustain resilience when facing implementation challenges. This study offers insights into how leadership development programs grounded in LHS principles could contribute to strengthening health systems in low‐resource settings.

The LLHS program shares similarities with established global health leadership programs [35, 36], including experiential learning, cross‐country collaborations, and peer networks. However, it is distinctive in its grounding in the LHS framework and its focus on both emerging and senior frontline clinical leaders, rather than primarily public health or research professionals. For example, five out of six global leadership programs reviewed by Rodríguez et al. targeted public health or research professionals, with only one including clinicians [35]. A systematic review similarly found that clinicians are under‐represented in LMIC leadership programs, possibly due to their perceived influence on system‐level changes [18]. Our program addresses this gap by equipping clinicians to lead health system improvement from within.

Participants reported a shift in their leadership identity, supported by two components: self‐reflective development of nontechnical competencies (such as emotional and social intelligence, empathy, and trust‐building) and the acquisition of practical leadership and quality improvement tools to support change. These competencies align with calls to integrate adaptive and relational skills, such as networking, communication, and collaboration, as key outcomes in clinical leadership development programs in LMICs [5, 18, 35, 37]. This study extends these findings to emphasize that relational skills are ideally combined with practical tools for change.

Participants demonstrated the ability to enact change at individual and team levels, such as fostering learning cultures. However, efforts to extend change beyond their circle of influence were constrained by environmental and social factors, such as hierarchical structure. These findings align with van Rensburg et al., whose mental health capacity‐building program in South Africa found that learning and implementation occurred mostly at the individual and team levels, with participants lacking formal authority to enforce organizational changes [16]. To counter the effect of hierarchical structures, participants described using relational mechanisms such as patience, relationship‐building, and peer networking. This reflects the concept of relational coordination, which emphasizes mutual respect and shared goals to reduce the disruptive effect of power on collaboration [38]. While the program could not alter local governance structures, it aimed to equip participants with relational capabilities to bridge formal authority and informal influence.

Participants from all five LMICs reported persistent resource constraints, including limited time, workforce, equipment, and funding. These challenges are well documented in the literature and often cited as barriers to health system improvement [3, 5]. While data and Health Information Systems (HIS) are central to LHS, underpinning the cycle from data to knowledge and improved practice [7, 39], their routine use in LMICs remains variable due to fragmented systems and limitations in the reliability, completeness, and usability of routinely collected data [3, 28, 40]. Participants demonstrated an emerging understanding of diverse data sources to inform improvement efforts, drawing on both electronic health records where available and locally accessible data such as audits, mortality reviews, and service‐level data. Notably, data availability or system maturity was not consistently identified as the primary barrier; instead, workforce and organizational constraints were more prominent. This aligns with literature highlighting technical, behavioral, and organizational factors influencing the routine use of data in LMIC [11]. These findings suggest the key challenge may not be the presence of data systems alone but the capacity of health professionals and organizations to engage with and act on available data.

In the face of persistent challenges, participants demonstrated a shift in mindset, recognizing that effective system change depends more on people than material resources. Rather than focusing on the barriers, they enacted adaptive leadership, reframing challenges as opportunities for learning and action. Strategies included fostering team‐based problem solving, using data to influence stakeholders and strengthening collaboration through shared resources. Participants in senior roles deliberately used positional authority to empower emerging leaders, connecting those with formal power to those motivated for change but lacking authority to act [5]. This approach aligns with calls to cultivate leadership early to support long‐term change [18]. Collectively, these behaviors reflect a shift from controlling resources to enabling people, consistent with adaptive, distributed leadership concepts that support resilience in complex health systems [41].

Our program adopts a bottom‐up approach by equipping local leaders, both senior and emerging, with tools and mindset to self‐initiate improvements based on LHS principles in their own setting. This contrasts with some LHS implementations in LMICs, where external agencies or funders introduce interventions for local teams to implement [14, 17, 42]. While such approaches offer the potential for improvements, our program emphasizes local ownership and capacity building across all levels, both of which are critical to advancing LHS development in LMICs [3, 43, 44].

The program's cross‐country, cross‐institutional Community of Practice (CoP) functioned as a key mechanism to sustain leadership efforts in building LHS [45]. Grounded in social learning theory [46] and constructivism [29], it enabled learning through interaction and shared experience, allowing participants to exchange practical strategies and navigate challenges in resource‐constrained settings. Consistent with literature describing CoP as a source of resilience through shared vulnerability [47], participants reported reduced isolation and a strengthened sense of solidarity. Given the complexity of health systems where no two LHSs are identical [8], these findings suggest a regional network of LHS‐informed leaders can enable cross‐system learning and sustain peer support in LMICs [48].

Evaluation of long‐term outcomes was beyond the scope of this 1‐year study. However, many participants demonstrated meaningful contributions within their local settings (Table 7). As noted in the literature, attributing systems change to a single intervention, such as a leadership development program, is difficult within complex, adaptive environments [5, 18, 49]. This is because leadership is only one of many interdependent domains required to build an LHS [9]. Longitudinal research is needed to assess whether the shifts in leadership approach and locally led initiatives observed in this study translate into broader systems‐level change over time.

This study has several limitations. The study involved a small cohort of participants who were selected through a nomination process based on their leadership roles and perceived system‐level influence. This may have introduced selection bias. Some participants spoke English as a second language, which may have impacted their capacity to express themselves in interviews. Our inductive findings based on participants' self‐report may reflect a social‐desirability bias. Involvement of researchers in the design and delivery of the program necessitated a reflexive approach to ensure the validity of qualitative data interpretation. This was enabled by having an independent researcher (CvH) who was not involved in program design or delivery to check themes and interpretation. Our study focuses primarily on the continuous learning culture domain of the LHS framework, foundational to enabling iterative improvement. Future research could explore how the intent and cultural shifts described by our participants translate into actual system changes in LMIC contexts.

## 5 Conclusion

This study demonstrates how a longitudinal leadership program grounded in the LHS framework supported health professionals in LMICs to develop systems leadership identity and influence team‐level changes. By equipping participants with self‐reflective skills, practical tools, and ongoing support from a cross‐country network and program faculty, the program cultivated adaptive, distributed leadership to build resilience within a resource‐limited environment. Critically, a lack of mature data systems should not be seen as a barrier to adopting an LHS approach in countries with low resources—value lies in the effective use of relatively simple, locally available data for improvement. These findings inform future leadership and capacity‐building initiatives aiming to advance LHSs in LMICs. Future research should investigate whether these leadership and cultural shifts translate into longer‐term system changes.

## Funding

The authors have nothing to report.

## Ethics Statement

This study was approved by The Royal Children's Hospital Melbourne Human Research Ethics Committee (HREC/109282/RCHM‐2024).

## Conflicts of Interest

The authors declare no conflicts of interest.

## Data Availability

The data that support the findings of this study are available from the corresponding author upon reasonable request.

## Appendix A

**1. Survey A**

*Guided by Kirkpatrick's evaluation framework, this survey explores initial reactions (Kirkpatrick Level 1) and learning (Level 2)*.

**Overall program**

How valuable have you found the Leading in Learning Health Systems program?

- No value
- A little value
- Some value
- High value

Can you tell us 1–3 things you learned? [open ended]

Can you tell us 1–3 things you would apply to practice? [open ended]

**Leading**

How valuable was the course on “Leading”?

- No value
- A little value
- Some value
- High value

**Learning**

How valuable was the course on “Learning”?

- No value
- A little value
- Some value
- High value

**Helping systems learn**

How valuable was the course on “Helping systems learn”?

- No value
- A little value
- Some value
- High value

**Other items**

How valuable has it been interacting with colleagues from different countries?

- No value
- A little value
- Some value
- High value

Would you recommend this course to other people in your country?

- Yes
- No
- Maybe

Is there anything else you would like to tell us or recommend for the future? [open ended]

**2. Interview A**

*This interview was conducted after participants completed Survey A. In this interview, participants elaborated on their learning experience, addressing initial reactions (Kirkpatrick Level 1) and learning (Level 2)*.

**Interview questions:**

Overall feedback of learning experience.

- How did you find the LLHS Program so far?
- What do you think of the relationship between Leadership and Learning Health System?

Intention to apply learnings.

- What are the key learnings you remember from the 2‐week intensive course in Melbourne?
- After attending this intensive course, how would you change your practice? (individual level)
- What are 1–2 things you might change in your team, organization, or system? (team, organization level)

Cross‐country learning

- How valuable has it been interacting with colleagues from different countries?

Ongoing support

- What other support could be useful to enable you to effect changes short term, medium term, long term?

Final thoughts

- Is there anything else you would like to tell us or recommend for the future?

**3. Survey B**

*Guided by Kirkpatrick's evaluation framework, this survey explores whether and how participants applied their learning in practice (Level 3) and any observable organizational impact (Level 4)*.

**Change in Behavior**.

How often do you use what you learned in the LLHS program in your work?

- Rarely
- Most months
- Most weeks
- Most days

How confident do you feel in applying the knowledge and skills gained from the LLHS Program in your daily work?

- (Rate on a scale from 1 to 5: 1 = Not confident, 5 = Very confident)

Which specific skills or knowledge from the LLHS Program have you found most useful in your role? (Select all that apply)

- Leadership practice
- Leading team and change
- Self‐awareness
- Using data for improvements
- Conflict management
- Learning culture and innovation.
- Learning Health System framework.
- Interprofessional collaboration
- Other (please specify) [open ended]

**Facilitation of Broader Change**

Have you been able to influence the culture of your team towards continuous learning and improvement?

- Yes
  ○ If yes, please give an example: [open ended]
- No
- Unsure

What barriers, if any, have you encountered when trying to implement changes based on the LLHS Program? (Select all that apply)

- Lack of time
- Insufficient resources
- Resistance from colleagues
- Organizational constraints
- Other (please specify) [open ended]

**Impact and Outcomes**

Have you been able to influence your organization to adopt changes based on the LLHS Program?

- Yes
  ○ If yes, please provide example: [open ended]
- No
- Unsure

**4. Interview B**

*This interview was conducted after participants completed Survey B. In this interview, participants elaborated on the LLHS Program's influence on their practice (Level 3 Behavior) and any observable changes within their team or organizational context (Level 4 Results)*.

**Interview questions**

Can you tell me about what has happened with your work after you returned home from LLHS Program in Melbourne?

- Were there any changes you have made to your practice? Please describe.
- Were there any changes (e.g., quality improvement projects, clinical audits) you have led or been part of since? Please describe.

What has helped you apply what you learned or initiate changes?

What challenges have you experienced in applying learning or implementing any changes?

Can you tell me if and how the LLHS program has helped you or your organization?

What would you like to see from the LLHS program in the future?
